# Supplementary material for: Disturbance in the protein landscape of cochlear perilymph in an Alzheimer’s disease mouse model
Source: PLoS One. 2024 May 10;19(5):e0303375. doi: 10.1371/journal.pone.0303375 (PMC11086917; doi:10.1371/journal.pone.0303375)
Supplement: S1 File — (DOCX) [file pone.0303375.s001.docx]

**Data availability:** The raw data for the proteomic analysis are available at https://repository.jpostdb.org/entry/JPST002363

**Funding:** This work was supported by grants from the Ministry of Education, Culture, Sports, Science and Technology (KAKENHI: 21K06421, 23H04167), Inamori Foundation, Brain Science Foundation, Narishige Fund, and Takeda Science Foundation to D.I.; AMED-CREST (23gm1510004) and Moonshot R&D (JPMJMS2024) to H.H. The funders had no role in study design, data collection and analysis, decision to publish, or preparation of the manuscript.

**Competing Interest Statement:** The authors declare no competing financial interests.
